# Same/Different Concept Learning by Capuchin Monkeys in Matching-to-Sample Tasks

**DOI:** 10.1371/journal.pone.0023809

**Published:** 2011-08-16

**Authors:** Valentina Truppa, Eva Piano Mortari, Duilio Garofoli, Sara Privitera, Elisabetta Visalberghi

**Affiliations:** 1 Unit of Cognitive Primatology and Primate Center, Institute of Cognitive Sciences and Technologies, National Research Council (CNR), Rome, Italy; 2 Department of General Psychology, University of Padua, Padua, Italy; 3 University ‘La Sapienza’, Rome, Italy; Yale University, United States of America

## Abstract

The ability to understand similarities and analogies is a fundamental aspect of human advanced cognition. Although subject of considerable research in comparative cognition, the extent to which nonhuman species are capable of analogical reasoning is still debated. This study examined the conditions under which tufted capuchin monkeys (*Cebus apella*) acquire a same/different concept in a matching-to-sample task on the basis of relational similarity among multi-item stimuli. We evaluated (i) the ability of five capuchin monkeys to learn the same/different concept on the basis of the number of items composing the stimuli and (ii) the ability to match novel stimuli after training with both several small stimulus sets and a large stimulus set. We found the first evidence of same/different relational matching-to-sample abilities in a New World monkey and demonstrated that the ability to match novel stimuli is within the capacity of this species. Therefore, analogical reasoning can emerge in monkeys under specific training conditions.

## Introduction

The use of abstract concepts improves the ability to sort objects, events, and relations into common classes on the basis of shared perceptual, associative, and relational properties and to transfer knowledge to new stimuli or contexts. In humans, these capabilities underpin advanced cognitive skills such as analogical reasoning. This type of reasoning is often considered the highest form of conceptually mediated behaviour because it involves the ability to judge relations-between-relations, or second order relations [1]–[3]. Concept learning has been the subject of considerable interest in comparative cognition. Nevertheless, the extent to which this ability is present in nonhuman species and whether or not the underlying information processing strategies are similar across species is still controversial [4]–[6].

In comparative research, abstract concept learning has been mostly investigated by using same/different discrimination tasks, in which subjects had to judge whether two items are physically the same or different [7]. These tasks require subjects to judge attributes shared in common between stimuli to be compared, i.e. first-order relations. Typically, two (or more) figures are presented simultaneously and subjects are required to respond by pressing one of two response keys. One key is associated with the presence of identical figures (*same* condition) the other one with the presence of non-identical figures (*different* condition). Another paradigm used to assess same/different concept acquisition is the Matching-to-Sample (MTS) task, which allows the use of multiple levels of abstraction varying in relational complexity. On the one hand, the identity MTS (IDMTS) involves solving first-order relations by choosing which of two (or more) comparison stimuli is perceptually equivalent to the sample stimulus. On the other hand, the analogical or relational MTS (RMTS) involves second-order relations, i.e. relations-between-relations, regardless of perceptual similarity among objects composing different sets [7], [8]. In fact, it requires subjects to understand whether the relationship among attributes of objects belonging to one set is equivalent to the relationship among objects belonging to another set (e.g., sets of objects of the same shape), with objects belonging to different sets always having different shapes.

Individuals may learn to solve the tasks reported above in a way that applies only to familiar stimuli (i.e., by item-specific learning), or in a way that goes beyond the training stimuli and applies to novel stimuli (i.e., by relational learning). Only in the latter case it is possible to infer that abstract concepts have been acquired [9]–[11].

Previous experience improves animals' ability to use same/different concept in order to judge relations-between-relations. Acculturation has been considered one of the most relevant factors and it has been argued that only apes that have been language trained [12] or that have been provided with non-linguistic symbol systems [7], [13] can cope with tasks involving second-order relations. However, recent findings contradict this view since gorillas and orangutans without a history of symbol training are successful in RMTS tasks [14]. Moreover, increasing the number of stimuli and the number of items composing the stimuli strongly improves the acquisition of a relational ability. Monkeys and pigeons trained with a large number of stimuli show a good transfer performance to novel stimuli in simple discrimination tasks [15], [16] and in matching tasks [17], [18].

Perceptual constraints also affect relational learning abilities. Great apes are able to solve RMTS tasks using stimuli made of only two objects or figures [13], [14], [19], whereas for non-ape species multi-item conditions are easier. Baboons [20], as well as pigeons [21], solve a same/different RMTS task with 4 x 4 grids of 16 *same* and 16 *different* stimulus arrays. In addition, baboons' performance dramatically decreases and reaches the level of chance with 2-icon stimuli when the number of items composing the stimuli is gradually reduced. Fagot et al. [20], argued that the drop in performance observed in their study was due to the amount of *entropy*, i.e. the amount of perceptual variation within a display. In fact, the amount of variance between same and different displays is more evident when the stimuli contain more items and facilitate solution. Finally, high spatial proximity between parts composing the stimuli (i.e., colour patches) improves the accuracy of baboons in a same/different RMTS task [22].

The current study evaluates the ability of the New World tufted capuchin monkeys (*Cebus apella*) to learn *same* and *different* abstract concepts and to use them to solve a relational matching task that mirrored the tasks presented to apes [13], [19], Old World monkeys (macaques: [23]; baboons: [20], and pigeons [21]). Previous studies demonstrated that capuchins solved other relational matching tasks based on the above/below concept [24] and on the relative size of a set of 3-dimensional objects [25]. In both these studies, monkeys were previously trained to solve IDMTS tasks, in which they searched for perceptual equivalence between stimuli.

In particular, the aims of our study were to assess: (1) if capuchin monkeys previously trained to solve MTS tasks on the basis of perceptual similarity (i.e., IDMTS: [18]), are able to use same/different concept to solve MTS tasks on the basis of relational similarity (i.e., RMTS); (2) whether or not performance can be improved by increasing the number of training stimuli and/or by increasing the number of items composing the stimuli. In fact, in contrast with previous RMTS studies on monkeys where the number of elements within the stimuli was gradually decreased, we adopted an increasing-element approach.

## Materials and Methods

### Ethics Statement

The research protocol for this study was approved by the Italian Health Ministry (Central Direction for the Veterinary Service, approval n. 11/2011-C). Housing conditions and experimental procedures were performed in full accordance with the European law on humane care and use of laboratory animals and complied with the recommendations of the Weatherall report (The use of non-human primates in research). To increase three-dimensional space available to the animals, indoor enclosures were furnished with perches and ropes and outdoor enclosures were furnished with logs, branches and ropes. Moreover, the presence of natural substrates, including woodchips on the ground, served to promote monkeys' exploratory behaviours. All subjects were habituated to the experimental cage, the experimental routine and the experimenters.

### Subjects

The subjects were five tufted capuchin monkeys (*Cebus apella*), two males (Robot and Sandokan) and three females (Pippi, Carlotta, and Roberta). All subjects were adults (8–27 years old) born in captivity and hosted at the Primate Center of the Institute of Cognitive Sciences and Technologies, CNR, Rome, Italy. They lived in three groups, each housed in an indoor-outdoor enclosure (indoor: 5 m^2^ × 2.5 m high; outdoor: 40 m^2^ × 3 m high). Capuchins were individually tested in an adjacent experimental cage (0.76 m long × 1.70 m wide × 0.73 m high), that they could access through a sliding door. Each subject was separated from the group just before the daily testing session solely for the purpose of testing. The testing occurred between 10:30 a.m. and 4:00 p.m. Water was freely available at all times. Fresh fruit, vegetables and monkey chow were provided in the afternoon after testing.

All monkeys were already familiar with the matching-to-sample procedure because they had been tested in tasks involving categorisation of visual stimuli with a touchscreen based apparatus [18].

### Apparatus

**Figure 1:**
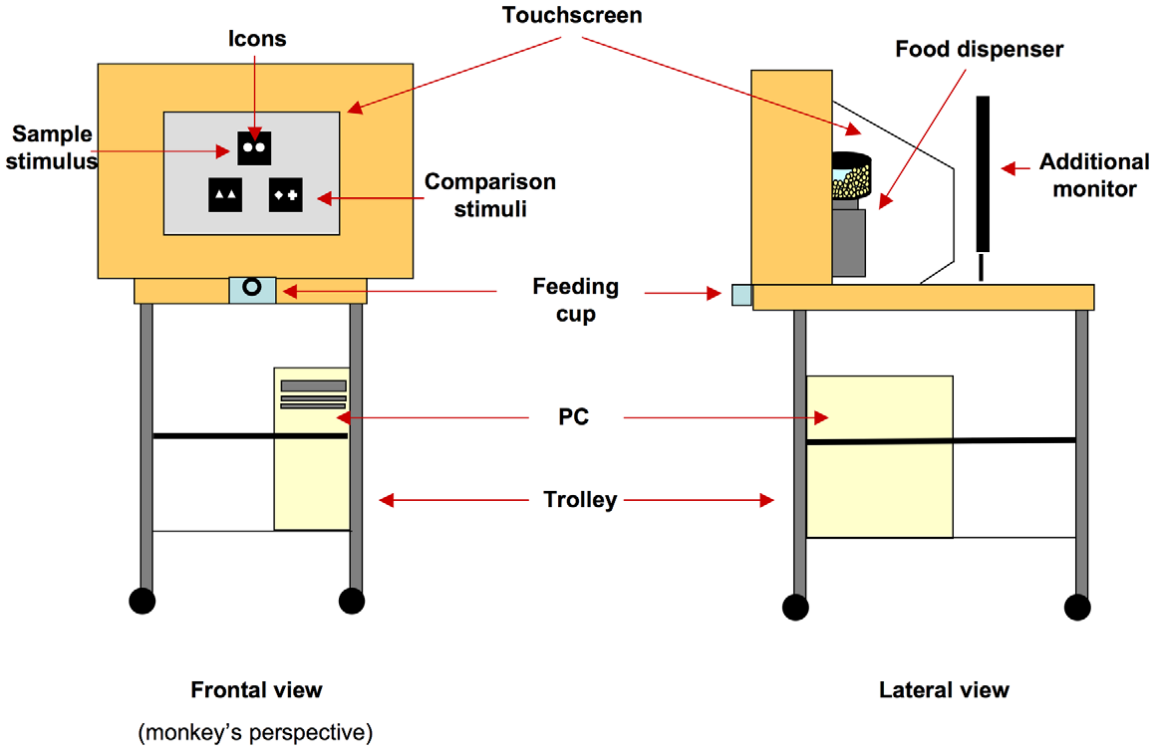
Experimental apparatus (modified from [**18**]).

The computerised test consisted of a PC (Model AMD Athlon 1200) connected to a 19” touchscreen (Model E96f+SB, CRT, ViewSonic) and an automatic food dispenser (Model ENV-203-45, MED Associates, Inc. Georgia, VT) (Figure 1). E-Prime software (Psychology Software Tools, Inc.) was used for the presentation of the stimuli and the recording of the subject's response. When the monkey provided the correct response, the food dispenser delivered 45-mg banana-flavoured pellets (TestDiet, Richmond, IN, USA) into a Plexiglass feeding cup (10 cm wide × 5 cm deep × 3.5 cm high) located 16 cm below the touchscreen in the centre.

A wooden frame (48 cm wide × 64 cm high × 30 cm deep) with a central aperture (36 cm wide × 26 cm high) surrounded the touchscreen. The food dispenser was placed behind the wooden frame, out of sight of the subject. Moreover, an additional LCD monitor was placed at the back of the touchscreen to allow the experimenter to see the progress of the session so as to remove the apparatus at the end of the session. The touchscreen, food dispenser and additional LCD monitor were mounted on the top shelf of a trolley (81 cm long × 45 cm wide × 80 cm high), whereas the PC was on the bottom shelf.

The apparatus was placed approximately 15 cm from the grid of the experimental cage within the arm's reach of the subject. The grid was made of horizontal metal bars (0.5 cm thick) that were separated by 4.5 cm.

### Stimuli

**Figure 2:**
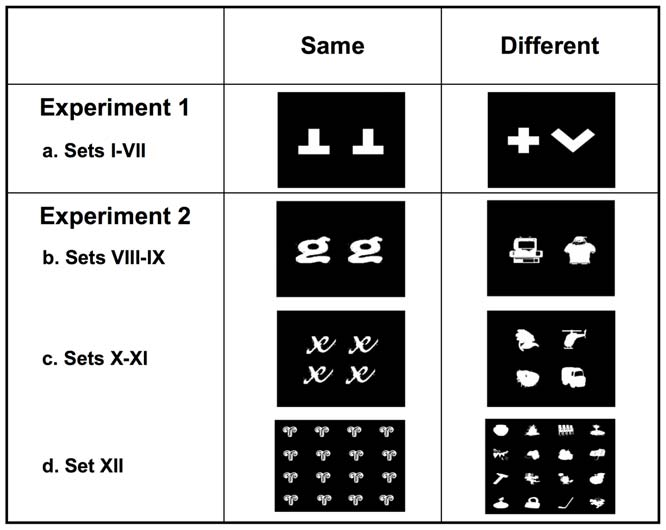
Examples of stimuli used in Experiments 1 and 2: (a) sets I-VII included four 2-icon stimuli, (b) sets VIII and IX included 288 2-icon stimuli, (c) sets X and XI included 288 4-icon stimuli, (d) set XII included 288 16-icon stimuli.

Each stimulus consisted of a black frame with two, four or sixteen icons inside (Figure 2). Twelve different sets of stimuli were used. Sets I-VII included four 2-icon stimuli, two stimuli had two identical icons and two stimuli had two non-identical icons (Figure 2a). A total of six white icons were used. Sets VIII and IX comprised 288 2-icon stimuli, 144 stimuli had two identical icons and 144 stimuli had two non-identical icons (Figure 2b). A total of 432 black and white icons were used. Sets X and XI included 288 4-icon stimuli (2 × 2 matrixes), 144 stimuli had four identical icons and 144 stimuli had four non-identical icons (Figure 2c). A total of 720 black and white icons were used. Set XII included 288 16-icon stimuli (4 × 4 matrixes), 144 stimuli had 16 identical icons and 144 stimuli had 16 non-identical icons (Figure 2d). A total of 2448 black and white icons were used. In sets I-IX each icon was on average 1.8 cm × 1.8 cm (6.8° of visual angle), in sets X-XI each icon was on average 1.5 cm × 1.5 cm (5.7° of visual angle), in set XII each icon was on average 1.2 cm × 1.2 cm (4.6° of visual angle). In sets I-XI icons were presented within 6.5 cm × 6.5 cm black frames, whereas in set XII icons were presented within 8.5 cm × 8.5 cm black frames. To increase the variability of icon spatial arrangements within the black frames, icons could be presented either aligned or misaligned on the vertical and/or the horizontal planes. All stimuli were made with computer icons and then converted into bitmaps before presentation on the computer screen.

### Procedure

A simultaneous Matching-to-Sample (MTS) procedure was adopted, in which three stimuli, the sample (SS) and the two comparison stimuli, the matching stimulus (S+) and the non-matching stimulus (S−), were presented on the computer screen. At the beginning of each trial, the sample stimulus appeared in the centre of the upper half of the screen. The sample consisted of identical or non-identical icons. Then, immediately after the subject touched the sample stimulus, the two comparison stimuli were simultaneously displayed on the lower part of the screen 5 cm apart from one another, and 4 cm below the sample (Figure 1). The positive (rewarded) comparison stimulus was the one presenting the same kind of relation (same or different) between/among its icons than the sample stimulus. The monkeys were required to touch the sample stimulus at the beginning of each trial in order to ensure that they were paying attention to it before the presentation of the comparison stimuli. The sample remained visible for the duration of the trial. The right/left positions of S+ and S- were randomly determined in each trial. The subjects had to indicate their choice by touching one of the comparison stimuli on the screen; the choice was automatically recorded by the computer. If the comparison stimulus was chosen correctly (S+), two food pellets were dispensed. If the selected stimulus was incorrect (S−), no pellet was dispensed. After the response, the display immediately extinguished and a 5-s inter-trial interval (ITI) followed a correct response, while both a 10-s time-out (TO) and a 5-s ITI followed an incorrect response. During the trials and the ITI the screen was light grey; during the TO the screen was green.

### Experiment 1

In Experiment 1, the subjects had to solve a same/different relational matching-to-sample task on the basis of the relation between the two icons composing each stimulus (same shape or different shape). Step-by-step we presented a total of seven small stimulus sets (Sets I-VII), each involving four 2-icon stimuli (two *same* stimuli including two identical icons and two *different* stimuli including two non-identical icons). A novel set was presented only after the monkey reached the learning criterion on the previous set. We hypothesised that the monkeys would require fewer trials to reach the learning criterion after the first set of stimuli. Moreover, on the basis of a previous study on identity concept acquisition [18] we expected capuchins to potentially show immediate transfer ability only after receiving several small sets of stimuli.

Initially, the monkeys were trained to match four stimuli from Set I. The training trial began with the presentation on the display of a sample stimulus and two comparison stimuli. The subject had to identify which of the two comparison stimuli included icons that had the same kind of relation featuring the sample (i.e., same icons or different icons).

At first, a correction procedure in which an incorrect trial was repeated until the subject made the correct response was adopted. Each session lasted until at least 48 correct responses were collected in which the four stimuli appeared as the sample an equal number of times. When the subject completed at least 48 correct responses (24 *same* and 24 *different*) out of 60 consecutive trials (corresponding to 80% correct responses), non-correction sessions were administered.

Each non-correction session consisted of 96 trials. Within the same session, the stimuli of Set I appeared as samples an equal number of times in a random order and each comparison stimulus appeared randomly at the left and the right of the screen with equal frequencies. When a subject met the predetermined criterion of at least 80% correct responses on two consecutive non-correction sessions both in the same and in the different trials, a transfer test was given.

Sessions were administered 5 days per week. The daily number of sessions varied according to the subject's motivation as well as the scheduled alternation of experiments of different studies in the experimental rooms. During correction training, subjects received 2–4 training sessions, whereas during non-correction training, subjects received 1-2 training sessions.

In the transfer tests, novel stimuli from Set II-VII were presented. Transfer tests consisted of two 96-trial sessions. In each session, to assess the extent to which the original matching performance was maintained half of the trials were based on a familiar stimulus pair and to assess the transfer half of the trials consisted of a new pair. Trials of the two types were randomly intermixed, and each comparison stimulus appeared randomly at the left and the right of the screen with equal frequencies. If the subject had an accuracy of 80% or more correct responses for the novel stimuli within the first 192 trials (that is over two sessions), a new transfer test was presented. Otherwise, the 96-trial sessions were repeated until the subject reached the criterion. This procedure was repeated for a total of six new sets of stimuli. Each transfer included a new set of stimuli and all subjects received the different sets in the same order. During transfer tests subjects received 1–2 sessions per day on 5 days per week.

### Experiment 2

As in Experiment 1, the subjects had to solve a relational matching-to-sample task based on the relation (same shape or different shape) between/among the icons composing each stimulus. Furthermore, in Experiment 2, we used large sets of stimuli (Sets VIII-XI) including either 2 or 4 icons. On the basis of previous studies, we hypothesised that concept acquisition would benefit from the use of large sets of stimuli [10], [15]–[18], [26], as well as from the use of stimuli with more than 2 icons [10], [20], [27]–[29]. In particular, we adopted an increasing entropy approach according to which the number of icons composing the stimuli rose from 2- to 4-icons if the subject failed with 2-icon stimuli. Therefore, monkeys were trained initially to match the stimuli made of two icons (Set VIII) and, if successful, received a transfer test with novel 2-icon stimuli (Set IX). If unsuccessful with 2-icon stimuli, they were trained and received a transfer test with 4-icon stimuli (Sets X and XI). Finally, the successful subjects with 4-icon stimuli were tested again with 2-icon stimuli and a novel condition with 16-icon stimuli.

Each new set of stimuli was introduced either when the subject performed significantly above chance (66.7%, p<0.05) in the *same* and in the *different* trials in the previous set, or when the subject performed 100 96-trial sessions without reaching criterion. Both in the training and transfer each session consisted of 96 trials (24 *same* trials aligned, 24 *same* trials misaligned, 24 *different* trials aligned, 24 *different* trials misaligned). Within each session the positive comparison stimulus (S+) appeared on the left and on the right with equal frequency. During training subjects received 1-2 training session for 5 days per week. Transfer tests consisted of one 96-trial session.

### Statistical analyses

The binomial *z* scores were used to assess whether or not the individual number of correct responses was significantly above chance (50%). The significance level was set at p<0.01 with small stimulus sets because in these sets the same stimuli were frequently repeated within a session increasing the probability of solutions due to item specific learning processes. In contrast, the significance level was set at p<0.05 with large stimulus sets because in these sets stimuli were never repeated within a session. Since the Kolmogorov-Smirnov test showed that the group data were normally distributed, parametric statistics were used to compare the accuracy scores across conditions.

## Results

### Experiment 1

**Figure 3:**
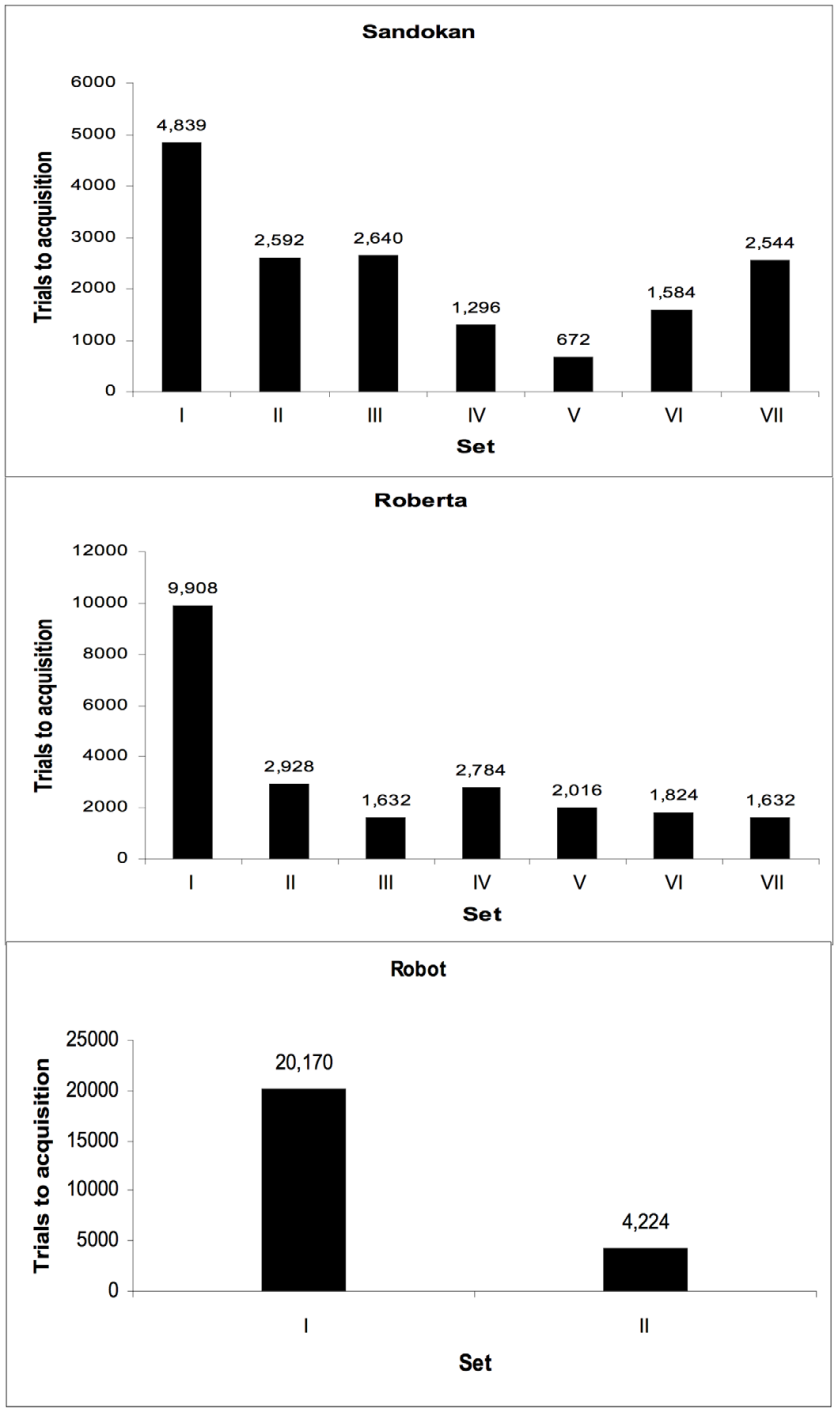
Experiment 1: number of trials to acquisition by each subject for each of the seven sets of stimuli.

Roberta, Sandokan and Robot completed both training with and without correction procedure, whereas Pippi and Carlotta completed only the correction sessions. Roberta and Sandokan received six sets of novel stimuli (Sets II-VII), whereas Robot received only one transfer test (Set II). Figure 3 reports the number of trials to acquisition by each subject for the first stimulus set and for each of the six following sets. Whereas Sandokan and Roberta received all six transfer tests, Robot received only one since his motivation decreased after the very high number of trials he went through to reach criterion in the first and the second set. There was great inter-individual variability in the number of trials required to reach criterion. With the first set of stimuli, to satisfy the learning criterion of 80% or more correct responses capuchins needed on average 11,639 trials (Sandokan: 4,839, Roberta: 9,908, Robot: 20,170), considering the sessions with and without a correction procedure.

After a total of 14,764 and 16,316 trials for Carlotta and Pippi, respectively, we interrupted their training because they never approached the learning criterion during training.

None of the subjects immediately transferred to the new sets of stimuli since they did not reach criterion within the first 192 trials. However, Roberta in the first session of Set VII was close to criterion. Her mean percentage of correct responses with the novel stimuli was 72.7 (*same*  =  75.0%, *different*  =  70.8%); this performance would have been above chance with an alpha level of 0.05.

Moreover, to assess whether the number of trials to reach criterion decreased between the first and the second sets of stimuli, we calculated the percentage decrease for the three subjects which completed Set I and Set II. On average, there was a decrease of 65.3% (Roberta, 70.4%, Sandokan, 46.4%, Robot, 79.0%; (Figure 3).

In the first training (Set I), there was a general advantage for the *same* over the *different* trials; all the five subjects had a significantly higher level of accuracy in the *same* (67.5%) than in the *different* trials (59.2%) [t (4) = 8.52, p<0.001]. And the same holds true for the three subjects that proceeded to the following sets, with the exception of Sandokan (Set IV) and Roberta (Sets III, IV, and VI).

### Experiment 2

#### Two-icon stimuli

All the five subjects completed the first 100 96-trial training sessions without reaching the criterion of 64.6% both in the *same* and *different* trials (p<0.05). All subjects did not perform higher than chance (the mean percentages of correct responses were: Carlotta, 49.2%, Pippi, 49.2%, Roberta, 49.5%, Sandokan, 49.2%, Robot, 49.3%). The percentage of correct responses was higher in the *same* trials (mean  =  54.5%, SD = 2.3) than in the *different* trials (mean  =  44.4%, SD = 2.4), [t (3) = 4.31, *p*<0.05] for all subjects except Sandokan.

#### Four-icon stimuli

**Figure 4:**
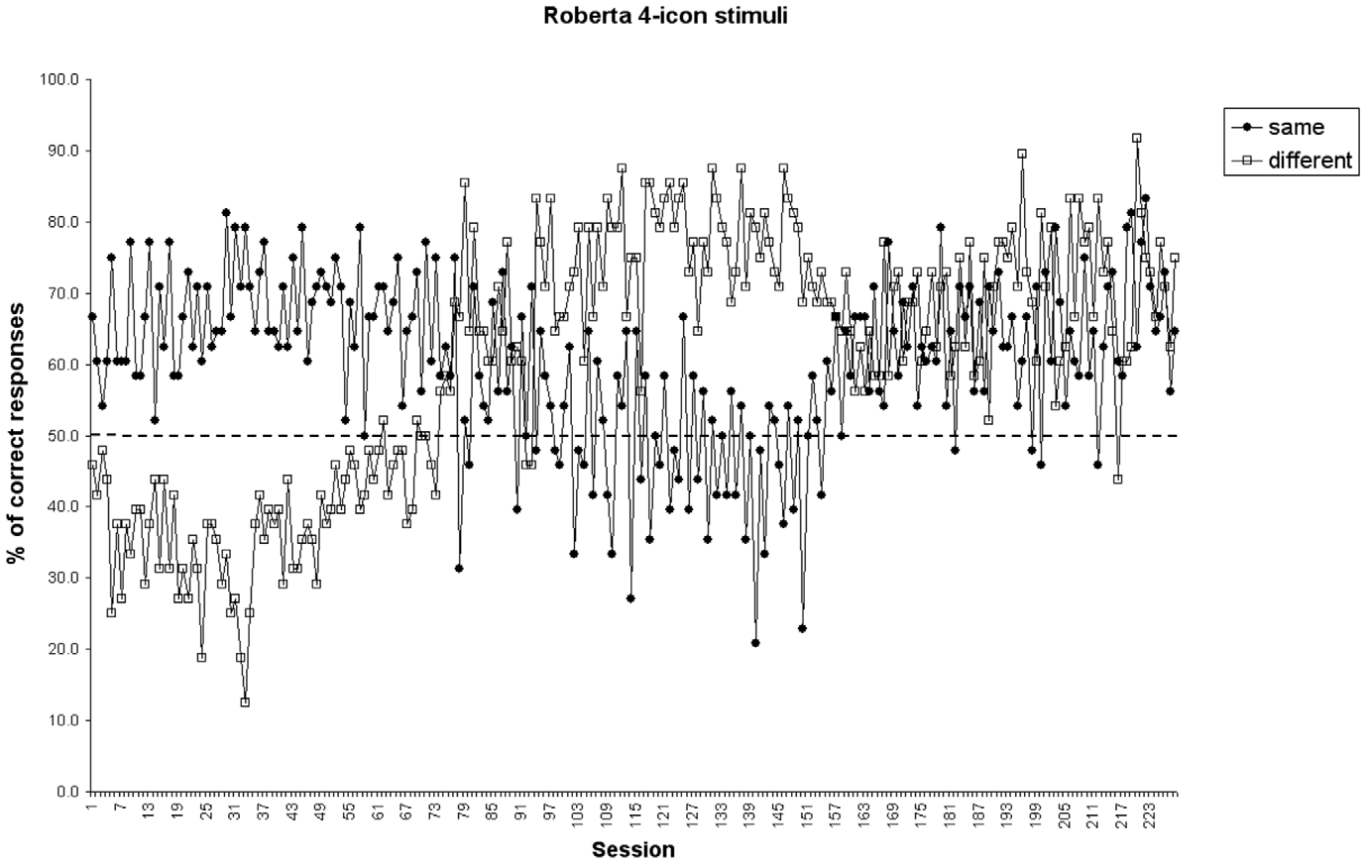
Experiment 2: percentage of correct responses of Roberta in the *same* trials (filled circles) and in the *different* trials (empty squares) in the 4-icon training condition.

**Figure 5:**
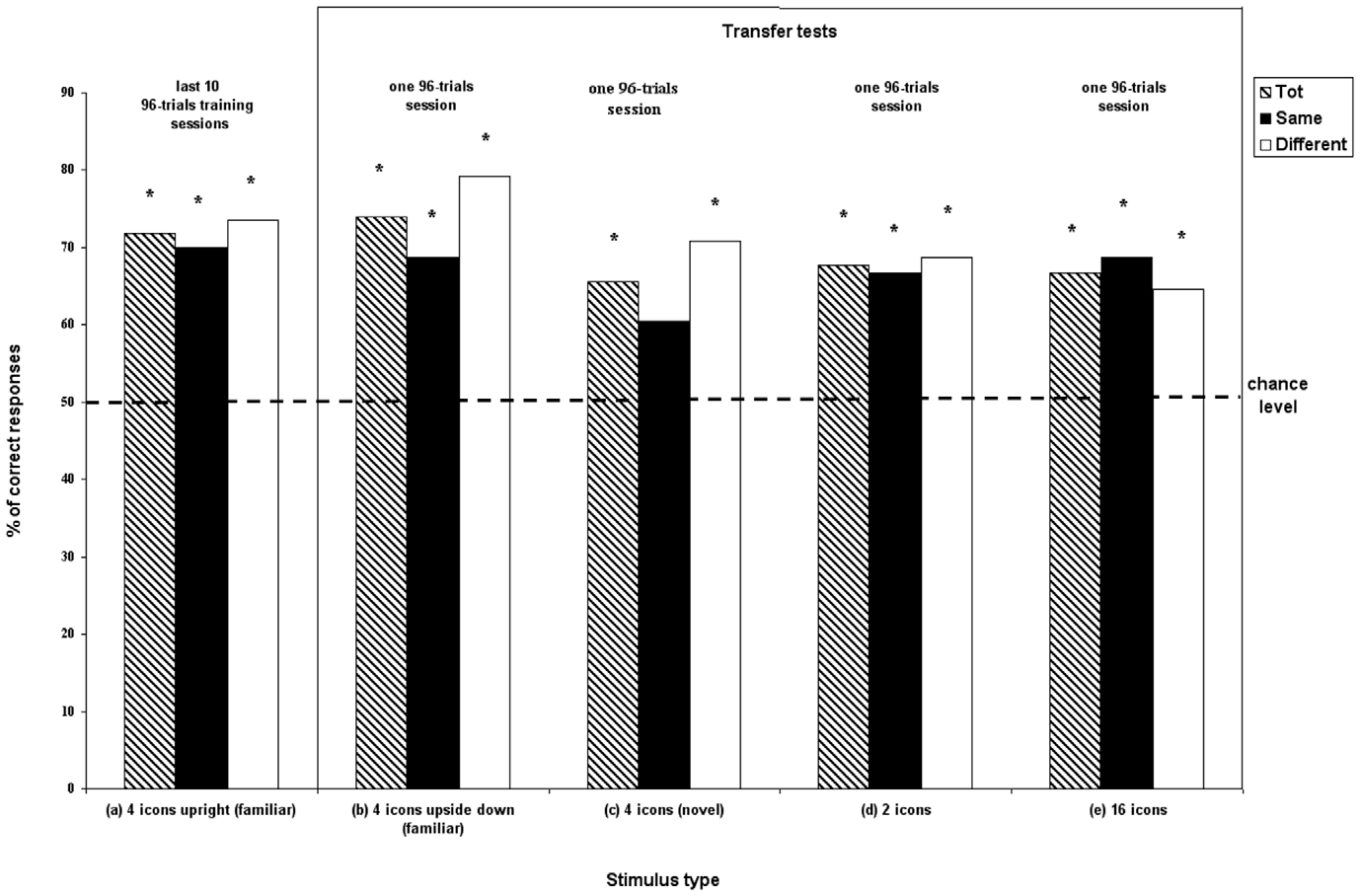
Experiment 2: Roberta's percentage of correct responses in (a) familiar 4-icon training stimuli, (b) familiar 4-icon stimuli presented upside down, (c) novel 4-icon stimuli, (d) 2-icon stimuli, and (e) 16-icon stimuli.

All the subjects completed the first 100 96-trial sessions without reaching the learning criterion (overall mean percentage: Carlotta, 50.7%, Pippi, 50.0%, Roberta, 54.8%, Sandokan, 50.5%, Robot, 51.1%). As in the 2-icon condition, Carlotta, Pippi, Roberta, and Robot showed a percentage of correct responses significantly higher in the *same* trials (mean  =  62.5%, SD = 7.5) compared with the *different* trials (mean  =  39.9%, SD = 3.0), [t (3) = 3.40, p<0.05]. After 77 96-trial sessions (7,392 trials) Roberta shifted from a pattern featured by high performance in the *same* trials and low accuracy in the *different* trials to a pattern with opposite trends. Hence, we kept her training during which she recombined the knowledge previously acquired and reached criterion in both types of trials (Figure 4). She took 228 96-trial sessions (i.e., 21,888 trials) to reach a performance stable around the 70% of correct responses both in the *same* and the *different* trials. At this point she received four transfer tests with different types of stimuli: (a) familiar 4-icon stimuli presented upside down, (b) novel 4-icon stimuli (video S1), (c) 2-icon stimuli (video S2), and (d) 16-icon stimuli (video S3). The performance of Roberta was significantly above chance in all the types of tests, with the exception of the novel 4-icon stimuli in the *same* condition, in which she did not overcome chance level for only two responses (familiar 4-icon stimuli upside down: mean Tot  =  74.0%, mean *same*  =  68.8%, mean *different*  =  79.2%; novel 4-icon stimuli: mean Tot  =  65.6%, mean *same*  =  60.4%, mean *different*  =  70.8%; 2-icon stimuli: mean Tot  =  67.7%, mean *same*  =  66.7%, mean *different*  =  68.7%; 16-icon stimuli: mean Tot  =  66.7%, mean *same*  =  68.7%, mean *different*  =  64.6%; Figure 5).

## Discussion

The current study demonstrates the acquisition of abstract concepts based on second-order relations by one capuchin monkey, Roberta. She was first successful with four-item stimuli and then with two-item stimuli, the latter being the most difficult condition previously thought to be mastered only by apes [7]. Since her performance was robust across different types of stimuli and well above that of the other subjects, we can argue that relational analogies are very difficult for capuchins, but under specific circumstances not impossible.

In Experiment 1, one capuchin lost interest in the task and received only two sets of stimuli and the other two capuchins failed to immediately transfer to novel stimuli after being presented with seven sets of stimuli, each including only four stimuli. Despite this poor performance, we cannot rule out the possibility that further reiteration of such a step-by-step procedure with small stimulus sets could eventually promote subjects' transfer ability and allow an immediate transfer to novel stimuli. However, our results seemed to be not promising in that sense. In fact, after the sharpest decrease occurring between the first and second sets, probably due to a ‘learning set’ phenomenon [30], many trials were always necessary to master the following five sets of stimuli. Our results suggest that capuchins solved the task by item specific learning every time they encountered novel stimuli. This finding contrasts with results on great apes demonstrating that chimpanzees [13], [19], gorillas, and orangutans [14], are able to transfer to novel stimuli after being trained with a limited number of exemplars.

In Experiment 2, using a large number of either 2- or 4-icon stimuli none of the five capuchins succeeded in reaching the learning criterion within 100 sessions (i.e., 9,600 trials). Furthermore, four out of five subjects performed significantly better in the *same* compared with the *different* trials. Similarly baboons tested with stimuli containing 2 or 4 icons, when a *different* sample stimulus is presented exhibit a strong tendency to choose the *same* comparison stimulus [20]. Also pigeons in a same/different discrimination task have a strong tendency to respond *same* with 2- and 4-icon *different* sample displays [28]. Likely, the low amount of variance in 2-icon *different* displays makes them similar to the *same* displays, thus increasing the percentage of *same* responses.

One capuchin (Roberta), after 228 sessions (i.e., 21,888 trials), succeeded in solving the 4-icon stimuli condition of Experiment 2 and in transfer to: (a) familiar 4-icon stimuli presented upside down, (b) novel 4-icon stimuli, (c) 2-icon stimuli, and (d) 16-icon stimuli. To our knowledge, this is the first New World monkey to solve the 2-icon condition in a same/different RMTS task. This result is consistent with recent findings demonstrating that one species of Old World monkeys, the Guinea baboons, solve the RMTS task when they judge stimuli made of pairs of colour patches [22]. The effect of the number of icons composing the stimuli on performance was already found in other species: pigeons [21] and baboons [20] can solve the RMTS tasks on the basis of the shape with displays including 16 icons, and baboons can transfer to displays with 12 and 8 icons, but their performance dramatically drops with 4-icon stimuli and was at chance level with 2-icon stimuli. Hence, performance worsens with decreasing entropy [20]. Since baboons were trained with decreasing entropy whereas capuchins with increasing entropy the finding that one capuchin succeeded with 2-icon stimuli supports the Flemming's [31] recent suggestion that monkeys tested with increasing entropy could be less dependent upon high levels of entropy. However, we cannot exclude that the higher number of trials that Roberta received accounted for her better performance. In fact, Fagot and colleagues [20] while training the baboons in the 2- and 4-icon stimuli conditions presented only 928 trials, a number of trials much lower than what we did.

Inter-individual differences are common in very cognitively demanding tasks. In a previous study, only one out of four capuchins solved a relational matching task based on the relative size of 3-dimensional objects [25] and not all the great apes individuals tested on RMTS tasks succeeded [13], [19]. Inter-individual differences may also lead to different strategies of solution; pigeons can learn an identical S/D task either by relational learning or by item-specific learning [32].

Our detailed analysis of the complete sequence of Roberta's training sessions highlighted a very distinctive learning trend. Initially, she seemed to spontaneously decompose the task in two sub-problems. Her learning pattern reveals that first she reached criterion on the *same* trials and then she reached criterion on the *different* trials. Her success in the *different* condition co-occurred with a worsening of performance on the *same* condition. Eventually, in the last part of the learning process, she recombined the knowledge previously acquired separately becoming concurrently successful in both conditions. On this basis we argue that a very demanding aspect, in terms of attentive resources and/or working memory load, of the same/different relational matching is to learn two concepts (sameness and difference) “at once”, that is when trials presenting *same* or *different* conditions are randomly alternated. If this were the case, presenting the *same* trials and the *different* trials separately should make the RMTS task less demanding than when the *same* and *different* trials are presented together. In this case, the solution of the RMTS task will require less parallel processing of information.

Roberta outperformed other capuchins also in other previous studies. In an IDMTS task Costello and Truppa [33] found a significant negative correlation between the overall performance of capuchins (in terms of number of correct responses across all training sessions) and the overall mean length of error sequences across blocks with an identical number of errors (from 10 down to 6 errors). This indicated that the better a capuchin's performance in the task, the more their pattern of response was consistent with the explicit hypothesis testing [34]. By testing an explicit hypothesis about which features of stimuli predict receiving a reward, a subject should adjust its choice on the basis of positive/negative feedbacks faster than by implicit associative learning. Consequently, when testing a hypothesis a subject should make shorter error sequences. In the IDMTS task Roberta did produce the shortest error sequences, associated with the best performance. Moreover, she outperformed other capuchins in a tool-using task consisting of a tube with a hole in the middle (the trap) with the reward placed nearer one of the two sides of the trap. The task involved second-order problem embodies two dynamic relations, one that the monkey must produce (between the stick and the food) and one that it must recognize (between the movement of the food over the trap and the food falling into the trap) [35]. Success can be obtained by avoiding the trap through a rule of action based on associative processes (such as push the food away from the trap, or insert the stick from the side farther from the reward) or by recognizing the outcome of pushing the food into the trap. In this task, Roberta, at that time only 3 years old, outperformed three older capuchins by adopting a distance-based rule of inserting the stick in the opening farthest from the reward. Interestingly for the present discussion, in the session before her systematic rise in success Roberta adopted the opposite strategy of erroneously inserting the stick from the side closer from the reward. Also in this case, her pattern of response seemed consistent with the explicit hypothesis testing.

The study of relational matching-to-sample across different animal species may prove to be a crucial step toward the comprehension of both abstract concept acquisition and the precursors of analogical reasoning. Our study indicates that same/different relational matching-to-sample abilities are within the ken of capuchin monkeys. Furthermore, it suggests that manipulating the number of figures composing the stimuli could facilitate the comprehension of second-order relations in capuchins, as argued for baboons [20]. Moreover, this study indicates that the most promising avenues for future research are the assessment of: (i) how inter-individual differences in learning strategies affect the acquisition of relational learning, (ii) the benefits of training subjects with a large variety of stimuli and/or with a very high number of trials, and (iii) the differences in relational learning acquisition across species.

## Supporting Information

Video S1 **Roberta, a female capuchin, carrying out the relational matching-to-sample task with 4-icon stimuli.**
(MPG)

Video S2 **Roberta, a female capuchin, carrying out the relational matching-to-sample task with 2-icon stimuli.**
(MPG)

Video S3 **Roberta, a female capuchin, carrying out the relational matching-to-sample task with 16-icon stimuli.**
(MPG)

## References

[1] Gentner D, Medina J (1998). Similarity and the development of rules.. Cognition.

[2] Vosniadou S, Ortony A (1989). Similarity and analogical reasoning..

[3] French R (1995). The subtlety of sameness: A theory and computer model of analogy-making..

[4] Penn DC, Holyoak KJ, Povinelli DJ (2008). Darwin's mistake: Explaining the discontinuity between human and nonhuman minds.. Behav Brain Sci.

[5] Wasserman EA, Young ME (2010). Same–different discrimination: The keel and backbone of thought and reasoning.. Anim Behav.

[6] Zentall TR, Wasserman EA, Lazareva O, Thompson RKR, Rattermann MJ (2008). Concept learning in animals.. Comp Cogn Behav Rev.

[7] Thompson RKR, Oden DL (2000). Categorical perception and conceptual judgments by nonhuman primates: The paleological monkey and the analogical ape.. Cogn Sci.

[8] Tomasello M, Call J (1997). Primate Cognition..

[9] D'Amato MR, Colombo M (1989). On the limits of the matching concept in monkeys (*Cebus apella*).. J Exp Anal Behav.

[10] Katz JS, Wright AA, Bodily KD (2007). Issues in the comparative cognition of abstract-concept learning.. Comp Cogn Behav Rev.

[11] Thompson RKR, Roitblat HL, Meyer JA (1995). Natural and relational concepts in animals.. Comparative approaches to cognitive science.

[12] Premack D, Premack AJ (1983). The mind of an ape..

[13] Thompson RKR, Oden DL, Boysen ST (1997). Language-naive chimpanzees (*Pan troglodytes*) judge relations between relations in a conceptual matching-to-sample task.. J Exp Psychol Anim Behav Process.

[14] Vonk J (2003). Gorilla (*Gorilla gorilla gorilla*) and orangutan (*Pongo abelii*) understanding of first- and second-order relations.. Anim Cogn.

[15] Katz JS, Wright AA (2006). Same/Different abstract-concept learning by Pigeons.. J Exp Psychol Anim Behav Process.

[16] Katz JS, Wright AA, Bachevalier J (2002). Mechanisms of same/different abstract-concept learning by rhesus monkeys (*Macaca mulatta*).. J Exp Psychol Anim Behav Process.

[17] Bodily KD, Katz JS, Wright AA (2008). Matching-to-sample abstract-concept learning by pigeons.. J Exp Psychol.

[18] Truppa V, Garofoli D, Castorina G, Piano Mortari E, Natale F (2010). Identity concept learning in matching-to-sample tasks by tufted capuchin monkeys (*Cebus apella*).. Anim Cogn.

[19] Flemming TM, Beran MJ, Washburn DA (2008). What meaning means for same and different: Analogical reasoning in humans (*Homo sapiens*), chimpanzees (*Pan troglodytes*), and rhesus monkeys (*Macaca mulatta*).. J Comp Psychol.

[20] Fagot J, Wasserman EA, Young ME (2001). Discriminating the relation between relations: The role of entropy in abstract conceptualization by baboons (*Papio papio*) and humans (*Homo sapiens*).. J Exp Psychol Anim Behav Process.

[21] Cook RG, Wasserman EA (2007). Learning and transfer of relational matching-to-sample by sample by pigeons.. Psychon Bull & Rev.

[22] Fagot J, Parron C (2010). Relational matching in baboons (*Papio papio*) with reduced grouping requirements.. Anim Behav.

[23] Flemming TM, Beran MJ, Washburn DA (2007). Disconnect in concept learning by rhesus monkeys (*Macaca mulatta*): Judgment of relations and relations-between-relations.. J Exp Psychol Anim Behav Process.

[24] Spinozzi G, Lubrano G, Truppa V (2004). Categorization of above and below spatial relations by tufted capuchin monkeys (*Cebus apella*).. J Comp Psychol.

[25] Kennedy EH, Fragaszy DM (2008). Analogical reasoning in a capuchin monkey (*Cebus apella*).. J Comp Psychol.

[26] Wright AA, Cook RG, Rivera JJ, Sands SF, Delius JD (1988). Concept learning by pigeons: Matching-to-sample with trial unique video picture stimuli.. Anim Learn Behav.

[27] Cook RG, Katz JS, Cavoto BR (1997). Pigeon same-different concept learning with multiple stimulus classes.. J Exp Psychol Anim Behav Process.

[28] Young ME, Wasserman EA, Garner KL (1997). Effects of number of items on the pigeon's discrimination of same from different visual displays.. J Exp Psychol Anim Behav Process.

[29] Young M, Wasserman EA, Hilfers MA, Dalrymple RM (1999). The pigeon's variability discrimination with lists of successively presented visual stimuli.. J Exp Psychol Anim Behav Process.

[30] Harlow HF (1949). The formation of learning set.. Psychol Rev.

[31] Flemming TM (2011). Conceptual thresholds for same and different in old- (*Macaca mulatta*) and new-world (*Cebus apella*) monkeys.. Behav Process.

[32] Elmore LC, Wright AA, Rivera JJ, Katz JS (2009). Individual differences: Either relational learning or item-specific learning in a same/different task.. Learn Behav.

[33] Costello F, Truppa V, Kokinov B, Holyoak K, Gentner D (2009). Individual differences in relational learning in capuchins: Evidence for hypothesis testing.. New Frontiers in Analogy Research: Proceedings of the Second International Conference on Analogy.

[34] Maddox W, Bohil CJ, Ing AD (2004). Evidence for a procedural learning-based system in category learning.. Psychon Bull & Rev.

[35] Visalberghi E, Limongelli L (1994). Lack of comprehension of cause-effect relations in tool-using capuchin monkeys (*Cebus apella*).. J Comp Psychol.

